# Electrochemically Synthesized MIP Sensors: Applications in Healthcare Diagnostics

**DOI:** 10.3390/bios14020071

**Published:** 2024-01-30

**Authors:** Akinrinade George Ayankojo, Jekaterina Reut, Vitali Syritski

**Affiliations:** Department of Materials and Environmental Technology, Tallinn University of Technology, Ehitajate tee 5, 19086 Tallinn, Estonia; akinrinade.ayankojo@taltech.ee (A.G.A.); jekaterina.reut@taltech.ee (J.R.)

**Keywords:** biomarker, disease diagnosis, molecularly imprinted polymers, electropolymerization, chemosensor, point-of-care testing

## Abstract

Early-stage detection and diagnosis of diseases is essential to the prompt commencement of treatment regimens, curbing the spread of the disease, and improving human health. Thus, the accurate detection of disease biomarkers through the development of robust, sensitive, and selective diagnostic tools has remained cutting-edge scientific research for decades. Due to their merits of being selective, stable, simple, and having a low preparation cost, molecularly imprinted polymers (MIPs) are increasingly becoming artificial substitutes for natural receptors in the design of state-of-the-art sensing devices. While there are different MIP preparation approaches, electrochemical synthesis presents a unique and outstanding method for chemical sensing applications, allowing the direct formation of the polymer on the transducer as well as simplicity in tuning the film properties, thus accelerating the trend in the design of commercial MIP-based sensors. This review evaluates recent achievements in the applications of electrosynthesized MIP sensors for clinical analysis of disease biomarkers, identifying major trends and highlighting interesting perspectives on the realization of commercial MIP-endowed testing devices for rapid determination of prevailing diseases.

## 1. Introduction

The modern healthcare sector grapples with a growing need for swift and dependable analytical techniques that can ensure optimal selectivity with a relevant limit of detection (LOD). In addition, such techniques should also be cost-effective, portable, and adaptable for real-time monitoring. Innovative biosensing strategies that allow biomarkers to be tested reliably in a decentralized setting are highly desired for replacing time-consuming laboratory analyses and for making analytical results available at the patient’s home (point-of-care testing, POCT) or through personalized health monitoring with wearable sensing devices [1,2]. POCT is essential for the rapid, early-stage detection of disease, which enables quick medical decisions and leads to improved health outcomes for patients. A major limitation of existing biosensing systems is the usage of unstable biological receptors as recognition elements. Although these receptors offer high selectivity for the target, they constrain the device’s shelf life and escalate its overall cost. Consequently, concerted efforts aimed at developing biomimetic substitutes for biosensors are on the increase [3,4].

Molecularly imprinted polymers (MIPs) are state-of-the-art synthetic receptors that combine robustness and high selectivity for a desired target analyte [5,6,7]. The predominant advantages of MIPs over biological receptors in sensing applications include fabrication simplicity, resilience in challenging experimental conditions such as varying temperature, pH, and exposure to organic solvents [8]. The integration of MIP-based recognition elements into various transducers, including gravimetric [9], field-effect transistors [10], optical [11], and electrochemical [12], has been intensively studied with the aim of developing medical diagnostic tools. Robust interfacing of a MIP layer with a transducer is a key aspect in the design of a MIP-based sensor. In terms of the deposition of MIP on a transducer, the polymerization method is accomplished by various synthetic routes, such as surface-initiated photopolymerization [13], solid-phase synthesis [14,15], the sol–gel approach [16,17], and electropolymerization [18,19,20]. Among these methods, electrosynthesis has shown outstanding benefits in terms of precise control of the deposition process, providing both a specified thickness and inner morphology of a polymeric material on the sensing surface of a transducer, as well as the feasibility of carrying out the synthesis at room temperature [18]. A survey of electrosynthesized MIP sensors for analysis of drugs, bioanalytes, proteins, etc. has been covered in several outstanding reviews [21,22,23,24,25].

This review presents an overview of the recent achievements in the field of electrosynthesized MIP-based sensors for in vitro medical diagnostics, covering the publications of the last 6 years, with a special emphasis on the application of such sensors for the analysis of disease-related biomarkers.

## 2. Electrochemical Synthesis of MIPs

Electropolymerization is a simple and widely used method for realizing MIP for sensing purposes and aiming at a reliable interface between the recognition element and the sensor transducer [21,26]. During electropolymerization, the voltage- or current-induced oxidation of the monomer is followed by the growth of the polymer film on the surface of the working electrode. The lack of external initiators, which may alter the structure of proteins, is a major advantage over chemically, thermally, or UV light-initiated polymerization. Another important advantage of the process is that the polymer thickness can be easily controlled by adjusting the amount of electric charge imposed on the electrode during electrodeposition, making the resulting MIP more reproducible than other polymerization methods. The fabrication of a MIP with the optimum thickness is especially crucial in the case of surface imprinting of macromolecules in order to avoid irreversible trapping of the template in the polymer as well as to provide sufficient charge transfer through the imprinted cavities to the electrode for electrochemical sensing of the target [27,28]. Moreover, the properties of the polymer coating, such as porosity and morphology, can be conveniently controlled by the appropriate selection of the experimental conditions, such as solution pH and electrolyte nature [18,29]. Finally, electropolymerization allows MIP synthesis in mild conditions, including aqueous media and room temperature, which is crucial when imprinting biological molecules, e.g., proteins. This, along with the absence of a requirement for toxic external initiators, presents electropolymerization as a green synthesis strategy. Notwithstanding, compared to chemically polymerized MIPs, the choice of electropolymerizable monomers is limited. Hence, electropolymerizable monomers such as pyrrole, aniline, thiophene, 3,4-ethylenedioxythiophene (EDOT), 3-aminophenylboronic acid (APBA), phenylenediamine (PDA), scopoletin, and dopamine (DA) are most often used. At the same time, the rational selection of an appropriate functional monomer is paramount for the success of molecular imprinting. Consequently, computer modeling has seamlessly been integrated into the design and optimization of MIPs, including electrosynthesized MIPs. There exists a plethora of MIP reports and reviews detailing the significance of computational methods and strategies for favorable MIP designs across diverse applications [30,31]. Depending on the choice of the monomer and the synthesis conditions, electropolymerization leads to the formation of either conducting or non-conducting polymer films. In contrast to the electrically conducting polymers that can be grown in thicker films, the growth of insulating polymer films is self-limiting in terms of thickness, resulting in thin films up to a few tens of nanometers. Sharma et al. published a comprehensive review on electrosynthesized MIPs for chemical sensors, with the principal focus on conducting and nonconducting polymers [21].

Biomarkers, serving as target analytes for MIP-based sensors, can be categorized into small organic molecules (<1.5 kDa) and biological macromolecules, e.g., proteins, based on molecular weight. Due to their unique peculiarities, the MIP preparation protocol to be employed should be given careful consideration. The easiest and most straightforward way to prepare MIPs for small analytes is the polymer film electrodeposition on the conducting surface of a sensor transducer from the electrolyte solution containing a monomer and a target analyte with the subsequent removal of the template either by washing with an appropriate solvent or by applying an electrochemical potential (dedoping) [32,33]. This method is referred to as bulk imprinting, where 3D binding sites are built for the entire molecular structure. However, achieving high sensitivity for small molecules may be challenging due to the comparatively lower response being induced by the sensor transducers. To address this, the possibility of designing MIPs at the nanoscale [14] as well as the incorporation of nanomaterials, e.g., carbon nanotubes (CNTs) [34], graphene [35], and metal nanoparticles [36], has been demonstrated. Nanomaterials bring several advantages to MIP sensing layers by enhancing the surface area-to-volume ratio, thus amplifying the number of binding sites and their accessibility to analytes [37]. On the contrary, imprinting of biomacromolecules, e.g., proteins, is more challenging due to their intrinsic properties, such as large size, structural complexity, and conformational flexibility [22]. A recent article provided a critical review of protein-MIP evolution as well as recent advances in the field [38]. The classical “bulk” imprinting strategy seems not to be a desired method for protein imprinting since it usually results in the complete entrapment of protein in the polymer, creating difficulties in its removal and subsequent rebinding. Nevertheless, there are several reports on successful bulk imprinting of protein biomarkers, including carcinogenic embryonic antigen (CEA), carbohydrate antigen 15-3 (CA 15-3), and 5-hydroxyindole-3-acetic acid (5-HIAA) [39,40,41].

Among a variety of approaches introduced to address the problem of macromolecular imprinting, surface imprinting has become the most popular, allowing the generation of binding sites at/or close to the polymer surface [42,43]. Thus, different surface imprinting strategies involving electropolymerization were developed to prepare MIP-based sensors for disease biomarker detection.

**Figure 1:**
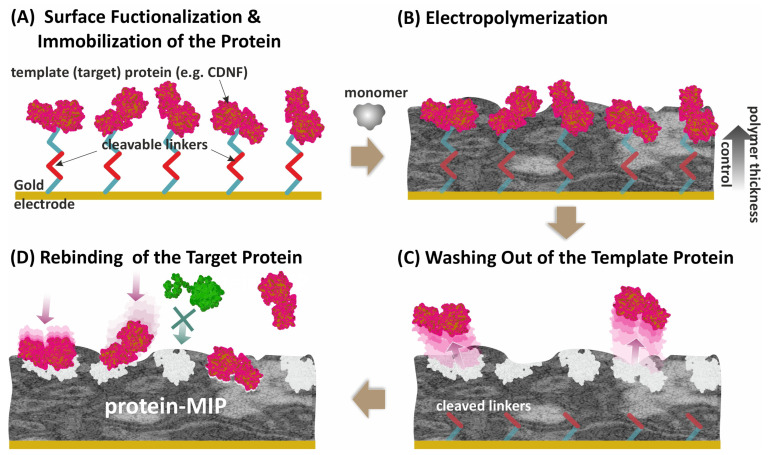
The surface imprinting strategy for the synthesis of a protein-MIP layer on a conducting surface using the electropolymerization approach. The strategy consists of (**A**) covalent immobilization of a target protein via a cleavable linker to a conducting surface; (**B**) electropolymerization of a monomer with careful control of the thickness of the growing polymer to exclude protein entrapment; (**C**) cleavage of the linker to facilitate protein removal; and (**D**) formation of MIP with protein-selective binding sites located on the polymer surface.

Syritski’s group developed an electrochemical surface imprinting strategy for target proteins that was successfully applied for imprinting immunoglobulin G [27], cerebral dopamine, and brain-derived neurotrophic factors (CDNF, BDNF) [44,45], and SARS-CoV-2 viral proteins [46,47]. The strategy consists of (A) covalent immobilization of a target protein via a cleavable linker to a conducting surface; (B) electropolymerization of a monomer with careful control of the thickness of the growing polymer to exclude protein entrapment; (C) cleavage of the linker to facilitate protein removal; and (D) formation of MIP with protein-selective binding sites located on the polymer surface (Figure 1).

**Figure 2:**
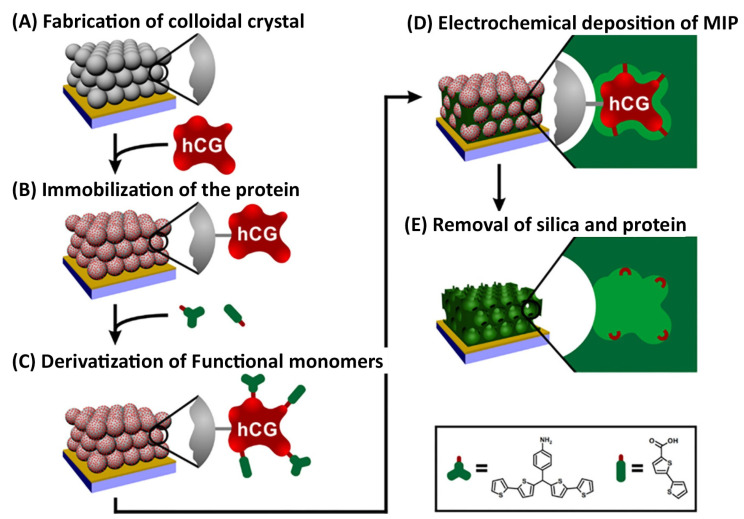
A surface imprinting approach combines colloidal crystal templating with electropolymerization to fabricate an inverse opal structure with molecular imprints of a protein (e.g., hCG) on the surface. The approach includes the following steps: (**A**) fabrication of a colloidal crystal template of SiO_2_ NPs; (**B**) immobilization of target protein (hCG) on the surface of NPs; (**C**) derivatization of protein with functional monomers (bithiophene); (**D**) electrodeposition of poly(2,3′-bithophene) film; (**E**) removal of NPs and the protein from the polymer, resulting in a polymeric inverse opal material with molecular cavities on the inner side of the pore wall. Reprinted with permission from M. Dąbrowski, A. Zimińska, J. Kalecki, M. Cieplak, W. Lisowski, R. Maksym, S. Shao, F. D’Souza, A. Kuhn, P.S. Sharma, Facile Fabrication of Surface-Imprinted Macroporous Films for Chemosensing of Human Chorionic Gonadotropin Hormone, ACS Appl. Mater. Interfaces. 11 (2019) 9265–9276. https://doi.org/10.1021/acsami.8b17951. Copyright 2023 American Chemical Society [49].

Polish researchers developed a surface imprinting approach by combining colloidal crystal templating with electropolymerization to fabricate an inverse opal structure with molecular imprints on the surface [48,49]. Thus, a hierarchical structure of a colloidal crystal template of SiO_2_ nanoparticles (NPs) was prepared using, e.g., the Langmuir–Blodgett technique (see Figure 2A). Then, template protein molecules (e.g., human chorionic gonadotropin (hCG)) are immobilized on the surface of these NPs (Figure 2B) and derivatized with functional monomers (bithiophene) (Figure 2C), followed by electrodeposition of poly(2,3′-bithophene) film in the hierarchical structure (Figure 2D). Finally, the removal of NPs and the protein from the resulting polymer leads to a polymeric inverse opal material with molecular cavities present exclusively on the inner side of the pore wall (Figure 2E). The developed approach resulted in a highly porous hierarchical MIP nanostructure that allowed the achievement of a remarkably high detectability of target protein.

Another approach to tackle the challenges of imprinting macromolecules is epitope imprinting, where a small surface-exposed fragment of a protein—an epitope—is used as a template [50,51]. Epitope templates, which are easily produced by artificial synthesis, are substantially more robust and stable than whole protein templates. In addition, as an epitope mimics the recognition domain of the antibody, epitope-imprinting would result in recognition sites that are more similar to natural ones, ensuring higher selectivity toward the target analyte [50]. Therefore, a MIP-based sensor developed on the basis of epitope imprinting was used for the recognition of neuron-specific enolase (NSE), a cancer biomarker [52]. Prior to imprinting, a computational simulation was employed to identify the most stable cysteine-modified secondary structure of the protein (Cys-Ep1) that was subsequently used as the epitope template. Electrosynthesis of a nonconductive film was achieved by cyclic voltammetry (CV) using scopoletin as the functional monomer. To reveal the binding cavities, the template was removed from the polymer by applying an anodic potential of 0.9 V. The authors later used the same approach but employed double-cysteine-modified peptides as the epitope templates that were immobilized on a gold surface by a self-assembled monolayer before electropolymerization [53].

## 3. Electrosynthesized MIP-Based Sensors for Detection of Disease Biomarkers

In this section, we will review reports that detail the electrochemical synthesis of MIP-based sensors designed for identifying biomarkers associated with various diseases. The focus will be on diseases commonly encountered in clinical settings, including cancer, cardiovascular problems, inflammatory disorders, neurological disorders, and infectious diseases.

### 3.1. Cancer

Cancer is a leading cause of death, accounting for one-sixth of the global mortality rate in 2020 [54]. Among the known types, lung, colorectal, liver, stomach, and breast cancers comprise the most common forms of cancer deaths [55]. There are several molecular biomarkers used across different stages of cancer progression that allow for the diagnosis and/or monitoring of treatment responses. Among other sensing materials, MIPs have gained research attention, demonstrating their potential to be employed as sensing layers in devices for cancer diagnosis [56,57]. Consequently, a variety of electrochemically produced MIP sensors are available for several biomarkers, such as CEA, carbohydrate antigens (CAs), epidermal growth factor receptor 2 (Her-2), NSE, 5-HIAA, etc.

**Figure 3:**
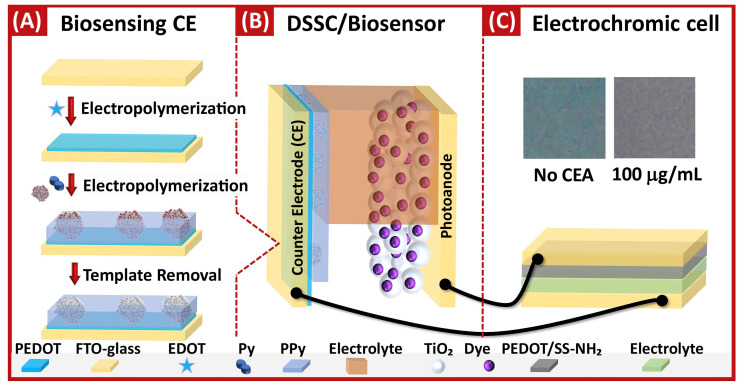
Scheme depicting multiple phases of preparation of a self-powered and self-signaled autonomous electrochemical biosensor for determination of CEA: (**A**) the assembly of the biosensing film on an FTO-glass substrate consisting of conductive PEDOT layer and CEA-selective MIP film; (**B**) the creation of the hybrid device by incorporating the MIP-modified electrode in a DSSC as the counter electrode; (**C**) the connection of the circuit with an electrochromic cell. The sensor undergoes a color change upon loading with CEA, transforming electrical energy into color via the electrochromic cell. Reprinted with permission from A.P.M. Tavares, L.A.A.N.A. Truta, F.T.C. Moreira, L.P.T. Carneiro, M.G.F. Sales, Self-powered and self-signaled autonomous electrochemical biosensor applied to carcinoembryonic antigen determination, Biosens. Bioelectron. 140 (2019) 111320. https://doi.org/10.1016/j.bios.2019.111320. Copyright 2023 Elsevier [63].

CEA is the most widely used tumor marker in clinical practice. It was found to be a useful marker for the diagnosis and prognosis of different cancers, including colorectal [58], lung [59], breast [60], and gastric [61] cancers. The accepted serum level of CEA is less than 3 ng mL^−1,^ and values above this could be indicative of cancer [62]. A self-powered and self-signaled autonomous electrochemical biosensor was designed to determine CEA [63] (Figure 3). The biosensor incorporates a photovoltaic cell, specifically a dye-sensitized solar cell (DSSC), where the biosensing occurs at one of the electrodes, namely the counter electrode (CE) of the DSSC. This electrode comprises a fluorine-doped tin oxide (FTO) glass substrate coated with a conductive poly(3,4-ethylenedioxythiophene) (PEDOT) layer and a CEA-selective MIP film (Figure 3A,B). The other electrode of DSSC was a traditional photoanode of titanium dioxide (TiO_2_) with a ruthenium-based dye and an iodide-based electrolyte. The concentration-dependent sensor response is generated by transforming electrical energy into color via an electrochromic cell consisting of a PEDOT/SS-NH_2_ electrochromic material, a semi-solid electrolyte, and an FTO-conductive glass (Figure 3C). The hybrid biosensor device exhibits a linear response in the range of 100 ng mL^−1^ to 100 μg mL^−1^ and a LOD of 0.14 ng mL^−1^ in human urine samples. Although a blood sample is typically used for CEA assessment, the result might suggest the sensor’s potential for a reliable investigation of the onset of the disease or to track the course of treatment.

**Figure 4:**
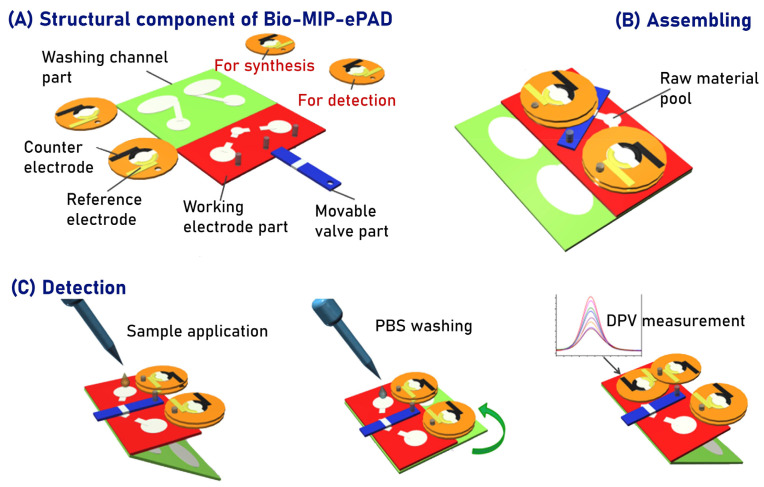
Schematic representations of (**A**) structural components of Bio-MIP-ePADs: the working electrode part (red), counter/reference electrode part (yellow), movable valve part (blue) and washing channel part (green); (**B**) fully assembled Bio-MIP-ePADs, featuring movable valves connecting the raw material pool and sample pools; (**C**) detection principle by the constructed Bio-MIP-ePADs consisting in application of sample to the working electrode pools, folding washing channels part and washing by phosphate-buffered saline (PBS) buffer solution following by opening another counter/reference electrode part and performing DPV measurements. Adapted with permission from J. Qi, B. Li, N. Zhou, X. Wang, D. Deng, L. Luo, L. Chen, The strategy of antibody-free biomarker analysis by in-situ synthesized molecularly imprinted polymers on movable valve paper-based device, Biosens. Bioelectron. 142 (2019) 111533. https://doi.org/10.1016/j.bios.2019.111533. Copyright 2023 Elsevier [64].

Qi et al. developed a sensor for the determination of CEA combining the surface imprinting technique and microfluidic paper-based analytical devices (Bio-MIP-ePADs) operating with a movable valve and origami method (Figure 4) [64]. The device comprises four parts: the movable valve, washing channels, working electrode, and counter/reference electrode parts. The counter/reference electrode part and the movable valve part were connected to the working electrode by rivets, while the washing channels were connected and utilized through origami folding. The design of the movable valve that was realized using hollow rivets as the holding center to control the channel of the ePADs in different layers has significantly enhanced performance during both synthesis and detection periods. The electropolymerization process for forming the MIP layer occurred on a microfluidic paper-based analytical device (μPAD), which had been previously modified with graphene oxide, chitosan, and CEA. As the fabrication protocol involved no external treatment and occurred solely on the μPADs, the resulting synthesis could be classified as green and offers both low cost and toxicity. The Bio-MIP-ePADs assessed by differential pulse voltammetry (DPV) displayed a linear range of 1–500 ng mL^−1^ with a LOD of 0.32 ng mL^−1^. In addition, the device’s performance, which was further tested in standard solutions of CEA within a concentration range of 0.6–5.0 ng mL^−1^, demonstrated agreement with the ELISA method. This was further established by comparing the analysis of real serum samples using Bio-MIPs-ePADs and chemiluminescent immunoassay, revealing no noticeable difference between the methods, with RSD ranging from 2.7% to 6.5%. Although the DSSC-based biosensor yielded a better limit of detection than in this report, it should be noted that the samples tested—urine and serum—have quite different levels of complexity, making a direct comparison between them a laborious task.

Furthermore, carbohydrate antigens such as CA15-3 and CA125 are also common cancer biomarkers for which MIP sensors have been fabricated. CA15-3 is extensively utilized as a serum marker for monitoring response or progression in metastatic breast cancer [65], while CA125 is linked to ovarian cancer [66]. For example, elevated levels of CA 15-3, i.e., concentrations above 30 U mL^−1^, are considered to be a potential indicator of breast cancer or its progression [62]. Thus, Pacheco et al. [67] developed a voltammetric MIP sensor for the analysis of CA15-3. The MIP production was based on direct surface imprinting of CA15-3 on the surface of a gold screen-printed electrode (Au-SPE) by electropolymerization of 2-aminophenol (2-AP). The voltammetric analysis was conducted using hexacyanoferrate (II/III) as a redox probe. A linear relationship between the sensor signal and the logarithm of the CA15-3 concentration was established between 5 and 50 U mL^−1^. The obtained LOD of 1.5 U mL^−1^ was well below the cut-off value in clinical practice (25 U mL^−1^). In another paper, CA15-3 was determined by an electrically conductive MIP-based electrochemical sensor fabricated using the electropolymerization of a water-soluble redox dye, toluidine blue, as a functional monomer on the Au-SPE precoated with a monomer-terminated SAM, thus ensuring the formation of a homogeneous and stable polymer film [40]. The calibration plots revealed a linear dependence of peak current over a wide CA15-3 concentration range (from 0.10 U mL^−1^ to 100 U mL^−1^) and low LOD (<0.10 U mL^−1^) in the buffer. Its performance was also validated in artificial serum. And yet, in another recent publication, CA15-3 MIP was fabricated on a paper-based carbon nanotube electrode that was previously modified with AuNPs. It was then modified by a MIP layer achieved by cyclic voltammetry electropolymerization of ortho-phenylenediamine (oPDA) in the potential range of −0.2 to 1.0 V at a scan rate of 50 mV s^−1^ and for 20 cycles. Although prolonged template elution and rebinding of the target were witnessed for about 2 and 1 h, respectively, the sensor reveals a good sensitivity of 0.013936 μA/U mL^−1^, a linear range of 5–35 U mL^−1,^ and a LOD of 1.16 U mL^−1^. Moreover, a satisfactory performance in serum samples was accompanied by good recoveries, suggesting that it is probably appropriate for use in the intended medium. However, a similar implementation of the sensor in the analysis of saliva samples fails due to low recovery and low reproducibility.

Another identified biomarker of breast cancer is the Her-2 protein, which was targeted by a MIP-based biosensing platform developed by using laser-scribed graphene (LSG) electrodes [68]. The electrodes were fabricated using polyimide by an irradiation CO_2_ laser, which was subsequently modified with nano-sized gold to increase its sensitivity and boost Her-2 immobilization. PEDOT was then electrodeposited around Her-2 by the chronoamperometry technique at +0.85 V prior to template removal in ethanol. The performance of the sensor, monitored by square wave voltammetry (SWV), showed promising results in recognizing Her-2 in the concentration range from 1 to 200 ng mL^−1^ and a LOD of 0.43 ng mL^−1^ below the cut-off value of 15 ng mL^−1^. Moreover, the sensor demonstrated a high selectivity for Her-2 against other interfering biomolecules and a good recovery (109–112%) in Her-2 spiked undiluted human serum samples. Finally, the POCT capability of the sensor was demonstrated by integrating it with a homemade electrochemical analyzer connected to the mobile analytical software.

NSE is a well-known marker of small cell lung cancer, and it is said to be the most reliable tumor marker in the diagnosis, prognosis, and monitoring of small cell lung cancer [69,70]. Tchinda et al. selected NSE as a template to develop biomimetic electrochemical sensors for the rapid detection of small-cell lung cancer [71]. The sensors were armed with scopoletin-based MIPs targeting NSE-derived peptide epitopes as well as the whole protein. The MIPs were synthesized via surface imprinting in the presence of cysteine- or histidine-modified epitope templates. These MIPs endowed the sensors with a selective ability to detect the peptide epitope in a concentration range of 2 to 128 µM and 15.6 nM to 128 µM, respectively, while the main target, NSE, could be detected in the range of 1 to 64 ng mL^−1^ and 0.25 to 64 ng mL^−1^, respectively. In a later report, the same research group succeeded in significantly improving NSE detection by introducing a fully electrochemical MIP sensor based on dual-epitope imprinting and gold nanoparticles (AuNPs) decoration achieved by hybrid epitope imprinting [72]. The developed sensor allowed the recognition of NSE in human serum in a concentration range of 25–4000 pg/mL with negligible cross-reactivity with dopamine, bovine serum albumin, glucose, and elongated peptide. The sensitivity level was enhanced to a great extent (20-fold) as compared to single epitope NSE-MIP [71] and allowed the ultrasensitive recognition of NSE in human serum.

5-HIAA is the primary metabolite of serotonin; its quantification in body fluids is found to be of high clinical significance as its altered levels draw attention to a human physiology disruption and a possible neuroendocrine tumor development [73]. Polypyrrole (PPy) nanothin film having molecular imprints of 5-HIAA formed on a glassy carbon electrode (GCE) served as a sensor capable of rapid electrochemical detection of 5-HIAA with a LOD of 15 pM in Britton–Robinson buffer, which was maintained (3.8% decrease in sensitivity) after almost 20 days of use and regeneration [41]. Furthermore, the sensor was successfully validated for the detection of 5-HIAA in serum, urine, and plasma, showing an excellent recovery between 98.86 and 101.52%.

Prostate-specific antigen (PSA) is considered the primary biomarker used for the diagnosis and follow-up of prostate cancer patients [74]. Wang et al. engineered a PSA sensor using electrochemiluminescence (ECL). In order to achieve high selectivity and sensitivity, this sensor was endowed with dual recognition, as provided by the integration of both aptamer and MIP recognition elements [75]. The sensor preparation involved an AuNPs-modified electrode on which the aptamer was self-assembled, followed by the formation of a MIP membrane through electropolymerization of dopamine (DA), resulting in the creation of an aptamer-MIP sensor. When PSA molecules are bound to the imprinted cavities, it suppresses electron transfer, causing a reduction in the ECL signal of luminol–H_2_O_2_ in the solution. This method of detection led to a LOD of 3.0 pg mL^−1^ within the range 5 pg mL^−1^–50 ng mL^−1^. Furthermore, the sensor was validated in human serum samples, showing its consistency with results obtained through clinical detection of PSA and a recovery range of 97.30% to 104.49%. This underscores the aptamer-MIP sensor’s remarkable capability to mitigate interference in complex biological samples. Another prospective biomarker for prostate cancer is sarcosine, which is detectable in both serum and urine samples [76,77]. Nguy et al. have used sarcosine as a target analyte to develop an impedimetric MIP-based sensor. The MIP layer was formed by electrodeposition of an ultrathin poly(4-aminothiophenol) film on top of the AuNPs-covered carbon SPE. The prepared sensor could detect sarcosine in buffer solution in the linear range of 1 ng mL^−1^–1.6 μg mL^−1^ with LOD and LOQ values of 0.76 ng mL^−1^ and 1.0 ng mL^−1^, respectively. In addition, the sensor was able to discriminate between sarcosine and interferent amino acids: l-alanine and l-lysine [36]. Other recent publications on the detection of cancer biomarkers by electrosynthesized MIP-based sensors are included in Table 1.

### 3.2. Cardiovascular Disease

Cardiovascular diseases are usually life-threatening and account for a major cause of death globally. Fortunately, several biomarkers are released into the blood during cardiac-related damage or stress that help in either predicting the onset of cardiovascular events or identifying individuals with the risk of developing the disease, hence helping in commencing preventive pharmacotherapy [93]. A recent review evaluating the significance of MIP-based POCT systems for cardiac disease-related biomarkers was documented [94]. One of the potent biomarkers of cardiac diseases and acute myocardial infarction (AMI) is cardiac troponin, cTn (I or T), which has been referred to as the gold standard cardiac biomarker [95]. In healthy humans, the decision limit for the levels of cardiac troponins in the serum is as low as 0.01 ng mL^−1^ [96]. Understandably, the imprinting of cardiac biomarkers, especially cTn, has received greater research attention.

Yola et al. developed a MIP-based biomimetic sensor where PPy imprinted with cTnI was electrodeposited on a GCE previously functionalized with uniform-sized boron nitride quantum dots (BNQDs) [97]. BNQDs provided an enhanced surface area and improved conductivity for the sensor electrode. The sensor showed high selectivity and sensitivity, a wide linear range (0.01–5.00 ng mL^−1^), and a LOD (0.5 pg mL^−1^) that was lower than previously reported detection methods. Further studies in plasma samples indicated that the sensor was selective for cTnI against cardiac myoglobin, bovine serum albumin, or cTnT, and reusability of up to 25 times was achieved. Furthermore, norepinephrine, a new functional monomer, was employed in building up a cTnI-imprinted film combined with an optical sensing system [98]. The authors utilized epitope-imprinted SPR biosensors to achieve cTnI detection. They concluded that the increased hydrophilicity of MIP-based norepinephrine compared to DA reduces the non-specific adsorption of proteins stemming from hydrophobic interactions with the surface. However, the LOD value (8.9 ± 1.9 nM) obtained by the direct detection of the analyte on the MIP-SPR sensor was far from the clinical performance requested by the international guidelines, ca. 40 ng/L (0.45 pM). To address the sensitivity issue, the authors proposed an amplification strategy based on an anti-TnC antibody labeled with alkaline phosphatase that allowed for a lower LOD value in spiked buffer solution, as well as demonstrated a prospective application of the MIP developed on high-sensitivity colorimetric/fluorometric platforms, i.e., in ELISA-type format, taking advantage of enzyme amplification.

Other troponins, including troponin-T (TnT), have also been utilized as templates for preparing MIP-based electrochemical sensors. A polyaniline-based MIP was electrodeposited on a carbon SPE modified with multi-walled carbon nanotubes (MWCNTs) and polymethylene blue (PMB) [99]. PMB was utilized as a redox-active layer and helped to avoid the need for a redox probe solution during rebinding. The sensor achieved a wide linear range of 0.10–8.0 pg mL^−1^ with a LOD of 0.040 pg mL^−1^ in buffer solution. The performance of the sensor in spiked human plasma was comparable with the gold standard electrochemiluminescence method in clinical use. In addition, an interesting approach to address the issue of decreased sensitivity in cTnT detection at low concentrations (<0.2 ng mL^−1^) by MIP-based electrochemical sensors was proposed [100]. The authors developed a new type of sensing electrode, an anodic aluminum oxide molecularly imprinted (MIP/AAO) nanocomposite electrode. MIP film was formed by electropolymerization of o-phenylenediamine (o-PDA) directly on AAO-modified electrodes, which can facilitate the vertical confinement of the redox current pathways to the bottom conductive electrode. With the addition of a one-dimensional AAO pillar, an improvement in the performance of the sensing electrode was achieved, resulting in a higher sensitivity of 1.08 × 10^–4^ ng mL^−1^ in the low-concentration regime (<0.03 ng mL^−1^) as well as a LOD value of 5.34 pg mL^−1^.

**Figure 5:**
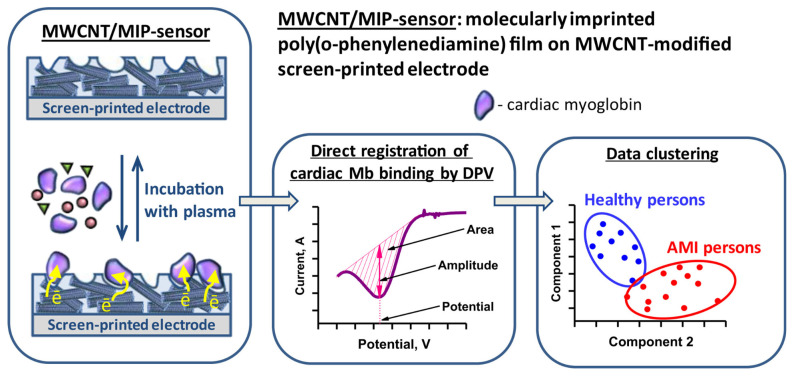
The arrangement of the MWCNT/MIP sensor, the principle of Mb detection, and the classification of plasma samples from healthy persons and patients with AMI. Due to the inherent redox property of Mb, the sensor can register it through direct electrochemical detection by DPV. Reprinted with permission from V.V. Shumyantseva, T.V. Bulko, L.V. Sigolaeva, A.V. Kuzikov, P.V. Pogodin, A.I. Archakov, Molecular imprinting coupled with electrochemical analysis for plasma samples classification in acute myocardial infarction diagnostic, Biosens. Bioelectron. 99 (2018) 216–222. https://doi.org/10.1016/j.bios.2017.07.026. Copyright 2023 Elsevier [103].

Other than troponins, myoglobin (Mb), a heme-containing redox protein, is considered one of the earliest markers of AMI, which can be used as an early confirmation of the disease onset [101]. Shumyantseva et al. reported on electrochemical sensors where a Mb-imprinted poly(o-PDA) was electrochemically synthesized on SPE [102] and MWCNT-modified SPE [103] (Figure 5). Due to the inherent redox property of Mb, its rebinding to a MIP sensor can be registered through direct electrochemical detection of the peak corresponding to Fe^3+^ reduction in the Mb molecule, thus avoiding the use of an external redox probe. In the first paper, MIP electrosynthesis was studied and optimized using the electrochemical quartz crystal microbalance (EQCM) technique, then integrated with SPE and tested in spiked human plasma, demonstrating a broad range of working concentrations of 1 nM to 1 µM and a LOD of 0.5 nM (9 ng mL^−1^), which is comparable with the sensitivity of immunosensors. In the next report, the researchers described a similar MIP-based sensor that was conjugated with MWCNTs to improve the sensitivity. The sensor was tested in the plasma samples of healthy people and AMI patients by applying a multi-parameter electrochemical analysis combined with subsequent computational clustering. This approach was found to provide better accuracy in the classification of plasma samples among groups of healthy people or AMI patients. Other recent publications on the detection of cardiovascular disease biomarkers by electrosynthesized MIP-based sensors are summarized in Table 1.

### 3.3. Inflammatory Disorders

Another prevalent clinical condition that has received MIP research attention is inflammation. They are accompanied by changes in the levels of certain biomolecules, e.g., interleukin-6 (IL-6) and 3-nitrotyrosine (3-NT), that are employed as biomarkers for identifying and monitoring the onset or progression of several inflammatory diseases.

To determine IL-6, a biomarker of brain inflammatory disorders [104], Gonçalves et al. developed a MIP-based electrochemical sensor by imprinting IL-6 in a poly(pyrrole-co-carboxylated pyrrole) electrodeposited on a carbon SPE [105]. The analytical performance of the sensor in an analyte-containing buffer and 100-fold diluted human serum was monitored by electrochemical impedance spectroscopy (EIS) and compared to the reference non-imprinted polymer-based sensor. The sensor displayed a LOD of 0.02 pg mL^−1^, which is lower than the cut-off level of 1.6 pg mL^−1^ in a healthy individual. Moreover, integrating the MIP with an SPE permits the POCT adaptation of the sensor and simple handling, thus indicating its suitability for clinical analysis. Also, a MIP synthesized on graphene quantum dots (GQDs)/functionalized MWCNT nanocomposite was adapted for determining IL-6 [106]. To form the composite, GQDs synthesized by oxidizing graphene sheets were mixed with MWCNTs and subsequently immobilized on GCE. Using CV, a PPy film was formed on the functionalized electrode in the presence of IL-6 as a template. In plasma samples, the electrochemical sensor exhibited a linearity range of 0.01–2.0 pg mL^−1^ and a LOD of 0.0030 pg mL^−1^, which demonstrates its superiority over those reported in existing literature for IL-6 determination. Moreover, the sensor was validated for high selectivity, stability, and good reproducibility.

In order to address the increasing need for wearable devices, Martins et al. [107] demonstrated a flexible electrical platform for the assembly of the electrochemical sensor targeted for 3-NT as a stable marker of oxidative stress in inflammatory diseases [108]. Practically, the sensor gold electrodes were fabricated on a transparent polymeric sheet substrate with a 3-NT-selective MIP layer assembled through electropolymerization of phenol in the presence of 3-NT. The sensor responses were derived by EIS, and under the optimized conditions, it was possible to detect 3-NT over the concentration range of 10 pg mL^−1^–1 μg mL^−1^ with a LOD of 1.13 pg mL^−1^, representing one of the lower LODs found in the literature. The authors foresee that such sensors can withstand mechanical deformations without compromising their electrochemical performance. Other recent publications on the detection of some inflammatory disease biomarkers by electrosynthesized MIP-based sensors are included in Table 1.

### 3.4. Neurological Disorders

Neurological disorders are malfunctions affecting the nervous system due to structural, biochemical, or electrical abnormalities in the brain, spinal cord, or other nerves of the human body [109]. Hence, to facilitate the achievement of curative or management goals of medical research via the provision of clinical evaluation and diagnosis, there is a need to design biomimetic sensors to detect known biomarkers of these disorders [110]. Neurotransmitters are responsible for the transmission of signals between neurons and non-neuronal body cells across chemical synapses. Abnormal transmission or variation in their concentration is directly associated with a variety of neurological disorders such as Alzheimer’s, schizophrenia, Parkinson’s, and depression [111]. Among neurotransmitters, DA, serotonin (SER), and epinephrine (adrenaline) are more commonly used as templates for MIP preparation [112].

**Figure 6:**
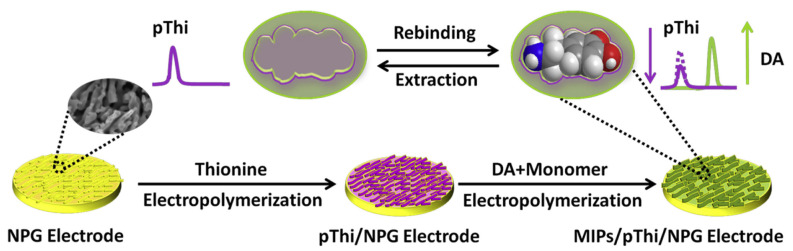
Schematic diagram of the MIP-based ratiometric electrochemical sensor for DA recognition. In the presence of DA in a solution, the sensor signals its presence by simultaneously decreasing the pThi- and increasing the DA-related DPV responses. Reprinted with permission from J. Yang, Y. Hu, Y. Li, Molecularly imprinted polymer-decorated signal on-off ratiometric electrochemical sensor for selective and robust dopamine detection, Biosens. Bioelectron. 135 (2019) 224–230. https://doi.org/10.1016/j.bios.2019.03.054. Copyright 2023 Elsevier [113].

By harnessing the benefits of the enlarged specific sensing area, Yang et al. prepared an electrochemical sensor where layers of polythionine (pThi) and MIP acting as the internal redox-active reference and DA receptor, respectively, were successively electrodeposited on nanoporous gold (NPG) [113]. Initially, the sensor featured a stable redox peak of pThi (a reference peak), which decreased after rebinding of DA, causing the appearance of an oxidation peak of the analyte on the sensing surface (Figure 6). The concentration-dependent response of the sensor is the ratio of DPV peaks for those processes. Thanks to the inner polythionine layer, the sensor is characterized by remarkable stability and the absence of analyte-independent interference of its responses in the broad dynamic range of 0.3–100 μM, as well as a low LOD of 0.1 μM. 

Yola et al. prepared an electrochemical sensor for SER using graphene quantum dots that are incorporated into 2D hexagonal boron nitride nanosheets [114]. Electrosynthesis of polyphenol in the presence of SER was achieved by CV, and the template was then washed out in a 1.0 M NaCl solution. The sensor showed better analytical limits (linearity range of 1.0 pM–10 nM and LOD of 0.2 pM) to SER than previous methods. Moreover, the target-selective response and the potential to analyze urine samples with good recovery were demonstrated. The unique electronic and catalytic properties of hexagonal boron nitride nanosheets, culminating in their stability, increased surface area, and conductivity, in addition to the selective properties of MIPs, contributed to the sensor’s performance. In another study, a nontoxic green functional monomer, nicotinamide, was employed in fabricating a MIP-based electrochemical sensor for epinephrine on a reduced graphene oxide modified GCE by CV [115]. The graphene-induced excellent electrocatalytic activity was displayed towards the target, resulting in improved sensitivity, high selectivity, a wide linear range (0.015 and 40 μM), a LOD of 3 nM, good reproducibility (RSD 3.44%), and good recoveries (96.0% to 100.7%) in urine and epinephrine ampoules.

**Figure 7:**
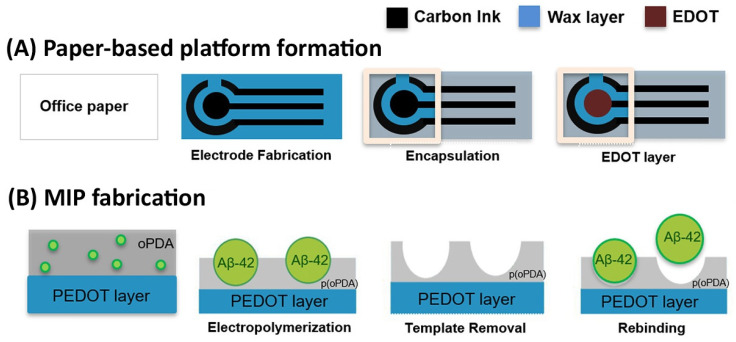
Schematic workflow for (**A**) carbon ink printed electrode formation on paper support and subsequent electrodeposition of the PEDOT layer; (**B**) MIP layer fabrication on top of the modified paper support. o-PDA is the monomer, and Aβ-42 is the target biomarker. Reprinted with permission from M.V. Pereira, A.C. Marques, D. Oliveira, R. Martins, F.T.C. Moreira, M.G.F. Sales, E. Fortunato, Paper-Based Platform with an In Situ Molecularly Imprinted Polymer for β-Amyloid, ACS Omega. 5 (2020) 12057–12066. https://doi.org/10.1021/acsomega.0c00062. Copyright 2023 American Chemical Society [120].

Apart from neurotransmitters, there are other specific biomarkers for different neurological disorders. For instance, α-Synuclein, a presynaptic neuronal protein, is genetically and neuropathologically linked to Parkinson’s disease, while amyloid-β protein is commonly associated with Alzheimer’s disease [116,117]. Thus, Ma et al. [118] reported a MIP-based electrochemical sensor for detecting α-Synuclein. To improve the sensor performance, a GCE was modified with a composite consisting of a nanospherical conjugated microporous polymer and graphene nanosheets prior to template immobilization and subsequent electropolymerization of pyrrole. Following template elution, the optimized sensor indicated a wide linearity (1 × 10^−4^ to 8 ng mL^−1^) and a low LOD (3.5 × 10^−5^ ng mL^−1^) comparable to other existing methods such as ELISA. Although its performance, tested against real patients’ samples, was yet to be evaluated, the sensor established a satisfactory capability in artificial serum samples. In another study aimed at fabricating a sensor for clinical diagnosis of Alzheimer’s disease, a known biomarker, amyloid-β 42 (Aβ-42) peptide, was the target [119]. Herein, delaminated titanium carbide (MXene) and MWCNT composites were combined with PPy-based MIP to enhance the sensor sensitivity towards the detection of Aβ42. The assay results in a linear range of two orders of magnitude and a LOD of 0.3 fg mL^−1^. Researchers from Portugal reported on the preparation of an eco-friendly and outstandingly inexpensive MIP-based electrochemical sensor for Aβ42 on a paper-based platform [120]. First, carbon-ink-printed homemade electrodes were fabricated on conventional office paper with subsequent electrodeposition of the PEDOT layer to improve the electrical features of the electrodes (Figure 7A). Then, a MIP layer was created on the carbon ink electrode’s surface by electropolymerizing o-PDA in the presence of Aβ42 and the subsequent removal of the template molecules by enzymatic and acidic treatments (Figure 7B). The sensor showed good operational characteristics in the range of 0.1 ng mL^−1^ to 1 μg mL^−1^ and a LOD of 0.067 ng mL^−1^ in spiked fetal bovine serum samples, as well as a fast response time (ca. 20 min).

In addition, neurotrophic factor proteins, e.g., cerebral dopamine neurotrophic factor (CDNF) [121], that have been shown to be a promising candidate for the treatment of Parkinson’s disease [122], could serve as potential biomarkers for early-stage diagnosis and/or follow-up of the neuroprotective therapy that is increasingly gaining the attention of the molecular imprinting research community. Thus, CDNF-specific nano-thin layers of MIP were electrogenerated on sensing elements of surface acoustic wave (SAW) transducers and adopted for ultra-sensitive detection of CDNF protein for early diagnosis of neurological disorders and monitoring of neuroprotective therapies [44]. By employing the surface imprinting strategy (Figure 1) involving the immobilization of the protein onto the electrode via a cleavable linker followed by electrodeposition of a layer of poly(m-phenylenediamine) (poly(m-PD)), CDNF molecular imprints were generated on the sensor substrate. The sensor depicted high discrimination between the target and its homolog in the sub-pg mL^−1^ range and a detection limit of 0.1 pg mL^−1^, illustrating its close competition with established protocols such as ELISA. Other recent publications on the detection of neurological disease biomarkers by electrosynthesized MIP-based sensors are included in Table 1.

### 3.5. Infectious Diseases

Despite the efforts of the global health system to promote human well-being, humanity has continually witnessed threats from chronic and emerging infectious diseases. Thus, disease outbreaks such as influenza, Zika, malaria, Ebola, dengue, Middle East respiratory syndrome, and the very recent severe acute respiratory syndrome, i.e., COVID-19, were prevailing global episodes over the last decades. The use of sensing devices targeted toward known biomarkers of these diseases is required for the early-stage identification and diagnosis of the infection and monitoring of treatment response [123]. A comprehensive overview of advancements in the development of POCT devices and MIP-based sensors for infectious diseases has been presented in several review papers [124,125,126].

To develop a biomimetic sensor for the diagnosis of liver infection, hepatitis C, a MIP-based biosensor for the recognition of hepatitis C virus (HCV) core antigen, was prepared by electropolymerization of DA, the functional monomer around HCV core antigen aptamer immobilized on GCE. To improve the conductivity, hence sensitivity, and aptamer loading capacity, GCE was modified with MWCNT-chitosan nanocomposite prior to aptamer immobilization and electrosynthesis [127]. To characterize the sensor, different electrochemical methods, including CV, DPV, and EIS, were used. The sensor indicated a linear response ranging from 5.0 fg mL^−1^ to 1.0 pg mL^−1^ and a detection limit of 1.67 fg mL^−1^. In addition, its practical application for the analysis of human serum samples was validated, thus suggesting the potential of the sensor to be used for the intended clinical analysis and subsequent diagnosis of hepatitis C infection. 

**Figure 8:**
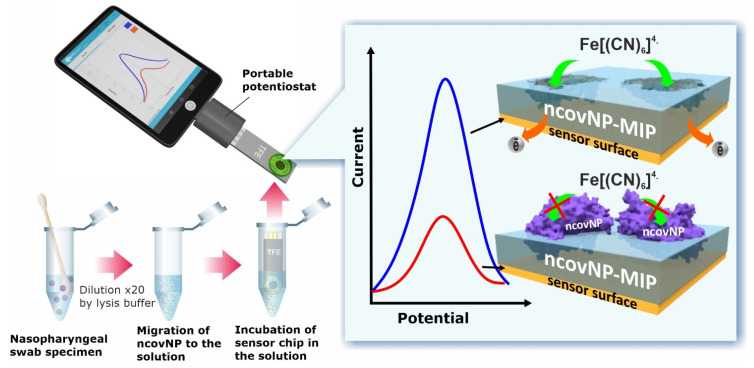
Principles of COVID-19 diagnostics using the SARS-CoV-2 nucleocapsid protein (ncovNP) imprinted sensor for analyzing samples prepared from nasopharyngeal swab specimens of patients. The Au-TFME, modified with ncovNP-MIP, is connected to a portable potentiostat. If ncovNP is present in the analyte solution, it occupies the imprinted cavities within the MIP film, resulting in a concentration-dependent reduction in the redox current peaks of the [Fe(CN)_6_]^3−^/[Fe(CN)_6_]^4−^ redox pair. The blue line represents the DPV current peak before incubation, and the red line represents the DPV current peak after incubation. Reprinted with permission from A. Raziq, A. Kidakova, R. Boroznjak, J. Reut, A. Öpik, V. Syritski, Development of a portable MIP-based electrochemical sensor for detection of SARS-CoV-2 antigen, Biosens. Bioelectron. 178 (2021) 113029. https://doi.org/10.1016/j.bios.2021.113029. Copyright 2023 [46].

Syritski’s research group published two reports where MIPs combined with gold thin-film metal electrodes (Au-TFME) were placed in service for express analysis of SARS-CoV-2 antigens as diagnostic markers of COVID-19 [46,47]. In the first report, SARS-CoV-2 nucleocapsid protein (ncovNP) was imprinted within poly(m-PD) by the surface imprinting approach, where noncovalent interaction played a major role in the analyte recognition (Figure 8) [46]. In the successive report, the group utilized SARS-CoV-2 spike protein subunit S1 to generate imprints in poly(APBA) by exploiting the covalent interaction between the boronic acid-containing monomer and 1,2-diols of the glycosylated target protein [47]. In both cases, DPV was applied to determine the analytical signals obtained from a decreasing current peak in response to an increasing analyte concentration. The sensors showed good characteristics, including a wide linear range and a low LOD in both the buffer and nasopharyngeal samples of COVID-19 patients, and results were comparable to PCR analysis, thus demonstrating the significance of the sensors for clinical analysis. It is important to highlight that the sensor delivered responses that can be easily measured with a portable potentiostat, thus holding significant potential for use in the design of a POCT platform for the swift and early diagnosis of COVID-19 patients.

In another recent contribution to the development of MIP-based electrochemical sensors for SARS-CoV-2 antigen detection, the research group of Ramanavicius imprinted SARS-CoV-2 viral spike glycoprotein in PPy [128]. Electrodeposition of the polymer on a platinum electrode was achieved by potential pulses from a phosphate-buffered saline (PBS) solution (pH 7.4) containing a mixture of SARS-CoV-2 spike glycoprotein and pyrrole. The study revealed enhanced glycoprotein rebinding as compared to the reference non-imprinted sensor, and selective recognition towards the target was demonstrated as against bovine serum albumin. Likewise, Tabrizi et al. imprinted a SARS-CoV-2-S receptor-binding domain (SARS-CoV-2-RBD) using CV-assisted electropolymerization of o-PDA in the presence of SARS-CoV-2-RBD on a macroporous gold SPE [129]. The impedimetric sensor displayed a good sensitivity and selectivity, a linear response of 2–40 pg mL^−1^, and a detection limit of 0.7 pg mL^−1^. Also, its clinical applicability was established by testing saliva samples spiked with different concentrations of SARS-CoV-2-RBD. An alternative approach for detecting SARS-CoV-2-RBD was introduced by Gyurcsányi’s group [130]. An alternative approach for detecting SARS-CoV-2-RBD was introduced by Gyurcsányi’s group. In this approach, the researchers engineered MIP-based microarrays selective for proteins on the surface of SPR chips. These microarrays were prepared by epitope imprinting a specific nonapeptide sequence, GFNCYFPLQ, unique to SARS-CoV-2-RBD, into polyscopoletin spots (approximately 500 μm). With such a system, it is anticipated that enlarging the number of elements in the microarray would facilitate its applicability for high-throughput screening of different virus variants as well as homologous and diverse protein targets within the limits of a single chip. Other recent publications on the detection of infectious disease biomarkers by electrosynthesized MIP-based sensors are included in Table 1.

### 3.6. Other Clinical Disorders

In addition to the disease biomarkers considered in Section 3.1, Section 3.2, Section 3.3, Section 3.4 and Section 3.5, for which MIP sensors are prepared, there are biomarkers for several other diseases that have also gained the attention of imprinting researchers. Dąbrowski et al. [49] applied the colloidal crystal templating strategy to fabricate surface-imprinted macroporous films for chemosensing of hCG hormone, which, in addition to the simple confirmation of pregnancy, can also be a marker of other pathological conditions like tumors [131]. The resulting macroporous MIP film integrated with electric transducers, namely, EG-FET and capacitive impedimetry, allowed the label-free, real-time determination of hCG hormone with detection limits of 0.8 and 0.17 fM as well as linear ranges of 0.8–50 and 0.17–2.0 fM, respectively, in 10 mM carbonate buffer (pH = 10). These are comparable with those parameters obtained from other biosensors reported for hCG analysis and cover the concentration range in which hcG exists in real patients’ samples.

**Figure 9:**
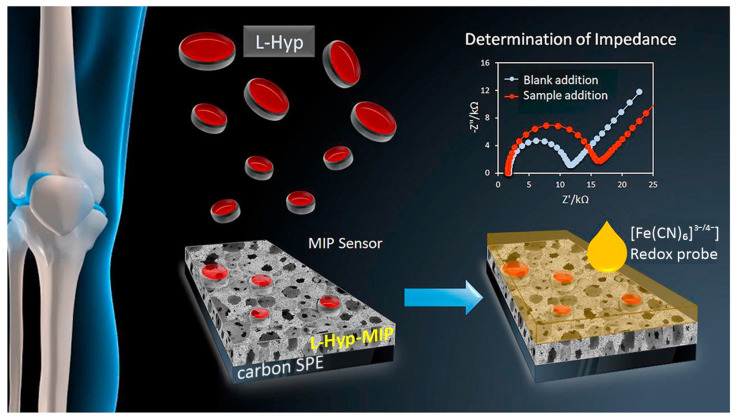
The measurement principle by enzyme-free impedimetric MIP-based biosensor for selective and sensitive determination of L-hyp. Reprinted with permission from W. Jesadabundit, S. Jampasa, K. Patarakul, W. Siangproh, O. Chailapakul, Enzyme-free impedimetric biosensor-based molecularly imprinted polymer for selective determination of L-hydroxyproline, Biosens. Bioelectron. 191 (2021) 113387. https://doi.org/10.1016/j.bios.2021.113387. Copyright 2023 [133].

An enzyme-free impedimetric MIP-based biosensor for selective and sensitive determination of L-hydroxyproline (L-hyp), a biomarker useful for early diagnosis of bone diseases, was reported [132]. Therein, a MIP layer was formed by CV on a carbon SPE by co-electropolymerization of APBA and o-PDA monomers in the presence of L-hyp as a template [133]. The sensor’s detection principle is based on changes in the charge transfer of redox probe ions [Fe(CN)_6_]^3−^/[Fe(CN)_6_]^4−^ through the imprinted cavities on the MIP upon rebinding of or in the absence of the target analyte, as measured by EIS (Figure 9). The sensor depicts a high sensitivity and selectivity towards the target with a LOD and linearity of 0.13 μg mL^−1^ and 0.4–25 μg mL^−1,^ respectively. Among other benefits, the fabricated biosensor offered several advantages, including simple operation, stability of up to 18 days, and successful implementation for L-hyp determination in human serum samples. 

In another study [134], by electropolymerizing a novel monomer, 2-amino-5-mercapto-1,3,4-thiadiazole, on a GCE electrode modified with reduced graphene oxide (rGO), a MIP layer was formed. The sensor was designed for the simultaneous detection of uric acid and tyrosine. Both are recognized as potential biomarkers for various disorders, including arthritis, neurological disorders, and kidney diseases [135]. The sensor’s principle entails the enhancement of the oxidation occurring on the MIP/rGO composite following the rebinding of the target molecules. After optimization, the sensor was capable of accurately detecting uric acid and tyrosine within a concentration range of 0.01–100 μM and 0.1–400 μM, respectively. In addition, detection limits of 3.2 nM and 46 nM were obtained for uric acid and tyrosine, respectively. Furthermore, the applicability of the sensor to detect the target analytes in serum and urine samples was demonstrated. Also, an electrochemical sensor for the determination of bilirubin, a biomarker whose concentration levels might be an indicative guideline for recognizing numerous liver diseases, was studied [136]. The sensor fabrication was achieved by electrodeposition of poly(o-PDA) film on an MWCNT-modified GCE from a solution consisting of o-PDA and bilirubin as monomers and template molecules, respectively. Bilirubin imprints were formed after acetonitrile-acetic acid treatment, which led to the elution of the template molecules. DPV revealed that the sensor not only had an excellent wide measurement range (12.1–91.8 fM) and a low LOD (7.8 fM) but could also recognize bilirubin in real samples, including the saliva and serum of infants, maintaining an acceptable shelf-life in terms of stability and sensitivity for over 10 days.

Another important target biomarker for which MIP electrochemical sensors have been developed is glucose. The changes in glucose concentration in the body fluid have attracted continuous attention for several decades because they are a fundamental part of the management of diabetes mellitus, a complex metabolic disorder with a rapidly increasing prevalence in the global population. Hence, the quantitative monitoring of blood glucose is of clinical importance and could greatly reduce the risks of diabetes mellitus-induced complications [137]. Thus, glucose-selective MIP-based sensors can provide an alternative non-enzymatic platform offering simple, cost-effective, and sensitive glucose monitoring systems [138]. Altintas’ group recently reported on the electrochemical glucose sensor based on AuNPs-decorated MIP. The sensor could detect glucose in human serum in a wide concentration range (1.25 nM–2.56 μM) with a detection limit of 1.25 nM. Moreover, the sensor could preserve its stability for up to around 95% during a storage time of 40 days [138]. In another report, a MIP-based electrochemical glucose sensor integrated with AuNPs’ modified screen-printed carbon electrode was developed for the determination of glucose in saliva and blood samples [139]. Using aminophenyl boronic acid as the functional monomer, reversible recognition of the analyte via boronate ester formation was achieved. The potentiometric sensor exhibited linearity ranging from 32 nM to 1 μM, a detection limit of 19 nM, and satisfactory performance in saliva and blood samples. Similarly, a glucose MIP was immobilized on the Au surface of SPE by the formation of a non-conducting polyacrylamide film using acrylamide/bis-acrylamide as the functional monomer. The sensor shows a linear dynamic range of 0.5 to 50 μg mL^−1^ and a detection limit of 0.59 μg mL^−1^. Moreover, the sensor displayed high selectivity and an analytical performance comparable to that of a reference glucometer for the analysis of saliva and blood samples [140]. Additionally, an innovative approach was employed, utilizing laser-engraved graphene, redox-active nanoparticles, and MIP-based receptors for the fabrication of a wearable electrochemical sensor. This sensor, equipped with in situ regeneration and self-calibration routines, enables real-time monitoring of trace levels of various biomarkers in human sweat, including essential amino acids, vitamins, metabolites, and lipids [141]. Other recent publications on the detection of other biomarkers (not covered in this section) by electrosynthesized MIP-based sensors are included in Table 1.

## 4. Conclusions and Future Perspectives

The availability of rapid and selective sensors is critical to the efficient running of the healthcare system and the early diagnosis of diseases. Molecular imprinting provides an attractive technique to create robust biomimetic recognition sites on sensor transducers that are adaptable for the targeted detection of disease biomarkers. Considering the biological origin of the target molecules, molecular imprinting by electropolymerization is highly advantageous since it allows MIP synthesis in mild conditions, e.g., aqueous media and room temperature. In this review, we assessed the diagnostic potential of electrosynthesized MIP-based sensors for various diseases by examining significant studies in the field. Particular attention was given to disorders such as cancer, cardiovascular, inflammatory, neurological, and infectious diseases due to their prevalence.

Analysis of the reviewed reports shows that most MIP-based sensors for healthcare applications utilized electrochemical transducers, mainly screen-printed and glassy carbon electrodes, integrated with a MIP film obtained mostly from the electrosynthesis of pyrrole as well as aniline derivatives. Such combinations are indicative of the potential of electrochemical transducers as the most suitable elements for the development of POCT devices. Moreover, the popular choice of pyrrole as the functional monomer for biomolecular targets is predictive since pyrrole presents excellent biocompatibility and ease of immobilization with different bioactive compounds [21]. The sensors evaluated in this review demonstrated sensitive and selective recognition of disease biomarkers and affirmed an obvious priority, namely, stability, over those equipped with natural recognition elements. Also, the applicability of the developed sensors in the intended biological matrices and at relevant concentration levels was established. Thus, the performance of electrosynthesized MIP-based sensors is attractive for the development of POCT devices for probing the onset or advancement of several diseases.

Although molecular imprinting technology has been around for more than two decades, the commercialization of MIP-based sensors still lags behind in comparison to sensors based on biological receptors [142]. The main bottleneck is the large-scale production of homogenous, high-affinity MIPs. However, a breakthrough solution, which was proposed by MIP Diagnostics Ltd. and pioneered by Piletsky’s group [143], focuses on MIP development using the solid-phase imprinting approach to producing high-affinity nanoparticles (nanoMIPs). Although, so far, a successful application of the nanoMIPs in combination with different sensor platforms for analyte detection has been reported [144,145], their robust integration with transducers still needs to be validated. Another issue in MIP large-scale production concerns protein imprinting since it requires a large amount of the template and its inherent costs. The epitope imprinting and solid-phase synthesis approaches could provide alternative strategies for cost-efficient protein-selective MIP fabrication.

Herein, we consider electrosynthesis to be an unparalleled route for accelerating the achievement of scalable mass production of MIP-equipped diagnostic devices. Firstly, electropolymerization could be classified as a green strategy for MIP synthesis due to the accommodation of mild synthesis conditions such as aqueous media, room temperature, and the elimination of toxic external initiators. Given the increasing concern for climate change and the demand for a greener world, the adoption of electrochemical synthesis within the MIP research community is expected to continue to expand. Moreover, development in sensor technology over the years has allowed for the mass production of low-cost, multiplex sensor elements, including SPEs and paper-based transducers that are compatible with electrochemical synthesis and analysis. Such elements, when combined with miniaturized electrochemical devices, e.g., a portable potentiostat driven by software on a mobile phone, could unlock a new route for the development of a valuable alternative diagnostic platform for the rapid screening of diseases. In fact, a breakthrough in this regard could engender the birth of smart, personalized medicine, where a preliminary screening conducted in the comfort of patients’ homes could be accessed directly and evaluated by the medical practitioner prior to the scheduled visit.

Although the difficulties of mass MIP production using electrosynthesis could be raised, we envision that engaging, for example, a microelectrospotting approach introduced by Gyurcsányi’s group [146] as a high-throughput method for the preparation of protein-selective MIP microarrays could be beneficial to circumvent such difficulties. In addition, the method matches the thrifty consumption of chemicals, which is especially advantageous in the case of protein templates. Moreover, microelectrospotting can also offer a means for multiplexed readout of MIP arrays, providing the possibility for rapid screening [147].

Furthermore, the potential for MIP-based sensors in wearable applications is substantial, converging electronics, materials science, and nanotechnology to create exceptionally effective, adaptable, and user-friendly systems for real-time and personalized monitoring. Modern, non-invasive devices for the real-time study of disease biomarkers may become possible by integrating MIPs with cutting-edge microfluidic chip technology, signal processing, and transmission electronics [141]. As technology progresses, this approach is likely to lead to more widely accessible and extensively used sensing applications. This strategy has already been applied in the detection of cortisol, a human stress hormone, and in glucose level monitoring [148,149].

In summary, there is enormous scope for the use of electrosynthesized MIPs as receptor layers for healthcare diagnostics. Currently, such layers, in combination with various sensor platforms, have demonstrated the capacity for sensitive and selective identification of biomarkers for many diseases at relevant concentration ranges, thus facilitating an early-stage diagnosis of a disease condition, thereby improving the quality of life and prolonging lifespan.

## Figures and Tables

**Table 1:** Electrosynthesized MIPs for sensing biomarkers for various diseases.

Target Analyte	Diseases	Monomer	Transducer	Sample	Linear Range	LOD	Ref.
CEA	Cancer (colorectal, lung, breast)	EDOT and pyrrole	Fluorine-doped Tin Oxide conductive glass substrate	human serum	0.1 ng mL^−1^–100 μg mL^−1^	0.08 ng mL^−1^	[78]
CEA	Cancer (colorectal, lung, breast)	DA		human serum	0.001–1000 ng mL^−1^	0.26 pg mL^−1^	[79]
CEA	Cancer (colorectal, lung, breast)	gallic acid	Au-SPE	PBS	1–100 ng mL^−1^	1 ng mL^−1^	[80]
CA 15-3	Cancer (breast)	o-PDA	Au-SPE	human serum	0.25–10.00 U mL^−1^	0.05 U mL^−1^	[81]
CA 15-3	Cancer (breast)	o-PDA	CNT electrode	human serum	5–35 U mL^−1^	1.16 U mL^−1^	[82]
TnT	AMI	o-PDA	Au electrode	human serum	0.009–0.8 ng mL^−1^	9 pg mL^−1^	[83]
DA	Neurological diseases	pyrrole	GCE	biological samples	62.5–100 μM	0.6 nM	[84]
PARK7/DJ-1	Neurological diseases	pyrrole	SPCE	lysis buffer	1–500 nM	1 nM	[85]
HCV-E2 ^a^	Hepatitis C	m-PD	Au SPE	human plasma	0.01–50 ng mL^−1^	0.46 pg mL^−1^	[86]
SARS-CoV-2 pseudoparticles	COVID-19	N-hydroxmethylacrylamide	Au-SPE	saliva	3.0–7.0 log10 pfu mL^−1^	4.9 log10 pfu mL^−1^	[87]
SARS-CoV-2 (ncovS1)	COVID-19	APBA	Au-TFME	nasopharyngeal swab	0–400 fM	64 fM	[47]
Glucose	Diabetes Mellitus	pyrrole	GCE	plasma	-	1 μM	[88]
3-nitrotyrosine	Oxidative stress	phenol	Carbon SPE	human urine	500 nM–1 mM	22.3 nM	[89]
3-nitrotyrosine	Oxidative stress	pyrrole	GCE	human serum and urine	0.2–50.0 mM	50 nM	[90]
Lactate	Hypoxia	APBA	SPE	sweat	3–100 mM	1.5 mM	[91]
Diosgenin	Diabetes	p-ABA ^b^	GCE	human plasma	0.003–0.13 mM	89.5 μM	[92]

^a^ Hepatitis C envelope protein E2 (HCV-E2), ^b^ para-aminobenzoic acid (p-ABA).

## Data Availability

No new data were created or analyzed in this study. Data sharing is not applicable to this article.
